# Clinical and Ethical Considerations in Allocation of Ventilators in an Influenza Pandemic or Other Public Health Disaster: A Comparison of the 2007 and 2015 New York State Ventilator Allocation Guidelines

**DOI:** 10.1017/dmp.2020.232

**Published:** 2020-07-14

**Authors:** Susie A. Han, Valerie Gutmann Koch

**Affiliations:** New York State Task Force on Life and the Law, Venture Catalyst, New York, New York; New York State Task Force on Life and the Law, New York, New York; MacLean Center for Clinical Medical Ethics at the University of Chicago, Chicago, Illinois; Health Law & Policy Institute at the University of Houston Law Center, Houston, Texas

**Keywords:** COVID-19/coronavirus, disaster planning, influenza pandemic, triage, ventilator

## Abstract

**Objectives:**

During an influenza or coronavirus disease 2019 (COVID-19) pandemic that results in acute respiratory distress, the number of available ventilators will not meet demand. In 2007, the New York State Task Force on Life and the Law and Department of Health released draft Guidelines for ethical allocation of ventilators for adults. In 2015, updated guidelines were released to ensure that: (1) revisions reflect the public’s values and (2) the triage protocol is substantiated by evidence-based clinical data. We summarize the development and content of the 2015 Guidelines compared with the 2007 version, emphasizing new/revised aspects of the ethical considerations and clinical protocol.

**Methods:**

We compared the 2007 and 2015 guidelines, with particular emphasis on the ethical issues and clinical protocols.

**Results:**

The 2015 Guidelines retained much of the ethical and clinical framework of the 2007 draft. The triage protocol was revised using evidence-based clinical data. Patients with the highest likelihood of short-term survival with ventilator therapy have priority access. Protocol consists of exclusion criteria, the sequential organ failure assessment (SOFA) score, and periodic clinical assessments. Guidance is provided on secondary triage criteria. Other forms of medical intervention/palliative care and review of triage decisions are discussed.

**Conclusions:**

The 2015 Guidelines reflect advances in medicine and societal values and provide an evidenced-based framework to save the most lives. The framework could be adapted in other emergencies, such as the COVID-19 pandemic, that require ventilators.

Since New York State released its draft ethical and clinical guidelines for the allocation of ventilators to adults in an influenza pandemic in 2007, public health emergencies, such as the novel H1N1 influenza pandemic in 2009, the Ebola virus disease in 2014, and now coronavirus disease 2019 (COVID-19), have increasingly captured the public’s attention. Early studies from COVID-19 cases in Wuhan, China, revealed that critically ill patients suffering from acute respiratory distress syndrome required ventilators.^1-3^ As of March 2020, northern Italy was experiencing widespread COVID-19 infections, making Italy the country with the second-highest number of infections after China.^4^ Hospitals in northern Italy were overwhelmed with patients with viral pneumonia and acute respiratory distress, and patients were being triaged by age and health status to determine who receives ventilator therapy and intensive care unit (ICU) beds.^5-7^ In addition, COVID-19 was also spreading rapidly in Europe.^8^ In late March 2020, the United States, specifically the New York City region, was an epicenter of the pandemic, accounting for approximately 5% of global COVID-19 cases.^9^ Similar to Italy, New York City is facing a bleak reality that soon there will not be enough ventilators,^10^ despite hospitals practicing “surge capacity” to reduce the need for ventilators by canceling or postponing elective procedures that require ventilators.^11^ Facilities are looking at New York’s ventilator allocation guidelines as a starting point for guidance on how to ethically allocate ventilators when demand exceeds supply.^10,12^

New York’s innovative 2007 Guidelines were among the first of their kind to be released in the United States and have been widely cited and emulated by other states.^13,14^ Since then, the New York State Task Force on Life and the Law (Task Force) and the New York State Department of Health (NYSDOH) have made extensive public education and outreach efforts and have solicited comments from various stakeholders. Following the release of the Draft Guidelines, at the request of the NYSDOH, the Task Force: (1) reexamined and revised the Adult Guidelines within the context of the public comments and feedback received, (2) developed separate guidelines for triaging pediatric and neonatal patients, and (3) expanded its analysis of the various legal issues that may arise when implementing the clinical protocols for ventilator allocation.^15^ This article summarizes the development and content of the revised 2015 Adult Guidelines compared with the 2007 Draft Guidelines, with emphasis on the new or revised aspects of the ethical considerations and clinical protocol, to assist other emergency planners when allocating scarce resources, especially during a respiratory-related pandemic such as influenza or COVID-19.

## BACKGROUND

The Guidelines recognize that patients generally expect to access or receive all necessary available health-care resources. However, in certain public health emergencies, these expectations may not align with the realities of the situation. The introduction of new technologies, medications, and interventions shift the public’s expectations of care. For example, during the 1918 influenza pandemic, there were no ventilators and the public expected treatment to be limited to addressing the symptoms of influenza. However, if an influenza pandemic of the same severity were to occur today, the public expectation will have shifted with the technology. With the World Health Organization’s (WHO’s) declaration that COVID-19 is a pandemic,^16^ the limited availability of ventilators will challenge the public’s expectation that patients should have access to all health-care treatments.

Recognizing that, in the event of a severe influenza pandemic, there would be more patients who require the use of ventilators than can be accommodated, the Task Force identified that the most obvious solution would be for facilities to stockpile more ventilators to address the shortage; however, such a proposal would be ineffective.^17^ First, there would still be an insufficient number of trained staff available to operate the ventilators and care for critically-ill patients. In addition, because facilities vary with regard to the models of ventilators they use, cross-facility use and training on how to operate the ventilators may be difficult. Finally, most ventilators are leased by facilities primarily because it is not cost-effective to own them. Because of these impediments to stockpiling a sufficient number of ventilators in preparation for a pandemic, a plan is needed on how to ethically allocate ventilators during an influenza pandemic.

## METHODS

The Draft Guidelines consisted of both nonclinical (ethical and legal) aspects as well a clinical protocol for ventilator allocation in an influenza pandemic. Between 2008 and 2015, the Task Force revisited the nonclinical aspects and added a more robust analysis of the ethical issues behind the triage protocol. The Task Force recognized that for the public to accept the Guidelines, they must reflect the values of New Yorkers. Thus, the Task Force oversaw a public engagement project in 2011, which consisted of 13 focus groups held throughout the state of New York. The Task Force also solicited public feedback on the Draft Guidelines through the NYSDOH website. To provide special expertise for the adult clinical protocol, in 2009, the Task Force convened an adult clinical workgroup comprised of individuals from the fields of medicine and ethics. The Task Force reviewed, deliberated on, and accepted the workgroup’s recommended refinements and revisions. As a result of these efforts, the 2015 Adult Guidelines incorporate comments, critiques, feedback, and values from numerous stakeholders and the public, including experts in the medical, ethical, legal, and policy fields.

The Adult Guidelines were written by Task Force staff and were reviewed and edited by the Task Force and clinical workgroup members. While the Adult Guidelines incorporate the same general framework as the earlier Draft Guidelines, several aspects were revised.

## RESULTS

The Guidelines affirm that efforts for surge capacity should be practiced by facilities to reduce the demand for ventilators when a pandemic is occurring. They should be implemented statewide by the appropriate governmental authorities and should only be followed as long as the circumstances require, with the understanding that pandemics do not necessarily follow a predicted trajectory and that resource availability can change. Statewide implementation is necessary to reduce inequalities of ventilator access and distribution among facilities and to ensure that the same resources are available and in use at similarly situated facilities.

In the event of an influenza pandemic necessitating implementation of the Guidelines, the clinical allocation protocol would apply to all patients in need of a ventilator, including those with conditions other than influenza. The ethical principles underlying the Guidelines—duty to care, duty to steward resources, duty to plan, distributive justice, and transparency—are preserved from the previous Draft. The Guidelines confirm that priority access to health-care workers, first responders, or other special groups should not be prioritized and reiterate that advanced age should not be a triage criterion.

Ventilators should be allocated to patients based on universally applied clinical criteria that evaluate a patient’s likelihood of survival. The use of a clinical evaluation system based on the Ontario Health Plan for an Influenza Pandemic protocol and on the sequential organ failure assessment (SOFA) score are maintained.^18-20^ The protocol is based on 3 steps: (1) application of exclusion criteria, (2) assessment of mortality risk using SOFA, and (3) periodic clinical assessments (time trials). Based on these clinical criteria, patients would be assigned a color code that determines their level of access to a ventilator (blue = lowest access/palliate/discharge, red = highest access, yellow = intermediate access, and green = defer/discharge). Under no circumstances should subjective or nonclinical factors, such as race, ethnicity, sexual orientation, socio-economic status, perceived quality of life, ability to pay, or status in the community be considered.

The primary goal remains to save the most lives in an influenza pandemic where there are a limited number of available ventilators. Patients for whom ventilator therapy would most likely be lifesaving are prioritized. A patient who is likely to survive with ventilator therapy is eligible for this treatment, and a patient who is unlikely to survive with ventilator therapy should instead receive other forms of medical intervention and/or palliative care so that another patient with a high likelihood of survival has an opportunity for ventilator therapy. A patient may only be removed from a ventilator after an official time trial clinical assessment reveals that such a step is appropriate. Ventilated patients in the blue color code category, and if there are no blue code patients, then patients in the yellow color category, are considered for other forms of medical intervention. In no circumstances should a red color code patient whose health status is improving at the time trials assessments be considered for ventilator removal. In addition, patients should not be compared with each other, because such comparisons may intensify inherent biases in the health-care system and the disproportionate and disparate provision of care for already disadvantaged populations.

Since the release of the Draft Guidelines, there was considerable discussion and public comment about the eligibility of ventilator-dependent chronic care patients to be subject to the triage protocol. While triaging these patients may increase ventilator supply, doing so relies on ethically unsound judgments based on subjective quality of life assessments. The Guidelines reaffirm that these patients should only be subject to the triage protocol when they arrive at an acute care facility, at which point they would be treated like any other patient who requires a ventilator. While this policy may deter chronic care patients from going to a hospital for fear of losing ventilator access, this policy balances the need to protect vulnerable populations with the principle of treating all patients in need of a ventilator equally. During a pandemic, long-term chronic care facilities should treat their patients as much as possible and only transfer patients to hospitals for serious and urgent conditions.

Several aspects of the Guidelines were revised from the Draft Guidelines (Table 1).

### Definition of “Survival”

Among the 2015 revisions, to bolster the goal of saving the most lives, the definition of “survival” is limited explicitly to a patient’s short-term likelihood of surviving the acute medical episode. This approach holds every patient to a consistent standard, and avoids subjective determinations of long-term survival, which are vulnerable to personal bias or quality of life opinions.

**TABLE 1:** Summary of Revisions Between the 2007 and 2015 Guidelines

Topic	2007 Guidelines	2015 Guidelines
Definition of “survival”	Not addressed	Defined to be a patient’s short-term likelihood of surviving the acute medical episode
Triage decision-maker	Triage officer	Triage officer or committee, depending on a facility’s resources
Exclusion criteria	Included medical conditions that required intense resources and those with high mortality, but ambiguous on when death would occur	Medical conditions limited to those associated with immediate or near-immediate mortality even with aggressive therapy (e.g., removal of renal dialysis as an exclusion criterion)
		Explicitly rejected as an exclusion criterion
DNR Order as exclusion criterion	Not addressed	
SOFA score	Provided cutoff scores for each color category with a brief explanation	More detailed explanation on how the scores are used and explains how a patient is eligible for/continues ventilator therapy, based on an improvement in overall health status (i.e., the SOFA score drops with each assessment)
Time trials	Time trials after 120 hours are not addressed	Assessments conducted every 48 h
Secondary triage factors	Not addressed	Use of randomization (e.g., lottery) or young age (i.e., 17 years or younger) may be used in limited circumstances
Alternative forms of medical intervention and palliative care	Briefly addressed	More detailed discussion
Review of triage decisions	Overview of both real-time appeals and retrospective review; did not recommend a model	Hybrid system of review, combining limited on-going individual appeals with retrospective periodic review

### Triage Decision-Makers: Triage Officer or Triage Committee

As in the Draft Guidelines, a separate decision-maker—not the patient’s attending physician—would be responsible for determining whether his/her patient receive ventilator therapy. Either a triage officer or a triage committee may be used to make the determination regarding whether a patient is eligible for ventilator treatment. Some facilities may prefer to have triage decisions be made by a group of individuals (triage committee). However, some rural facilities may not have the staff resources to convene a triage committee. The decision regarding whether to use either a triage officer or committee should be left to each facility based on differing available resources.

The use of a triage officer or committee will enable smooth and effective administration of the Guidelines by maintaining role sequestration. While the attending physician interacts with and conducts the clinical evaluation of a patient, the triage officer or committee does not have any direct contact with the patient. Instead, the triage officer or committee examines the data provided by the attending physician and makes the determination about a patient’s level of access to a ventilator.

### Revisions to the 3-Step Triage Protocol

Despite maintaining the 3-step triage protocol enunciated in the Draft Guidelines, several aspects of the protocol were revised and refined to ensure that inclusion of a triage criterion was substantiated by evidence-based clinical data. In addition, the Guidelines explicitly state that the adult protocol applies to individuals age 18 and older.

#### Step 1: Exclusion Criteria

The list of medical conditions that qualify as exclusion criteria was revised and limited to those associated with immediate or near-immediate mortality even with aggressive therapy (Table 2). Many of the medical conditions listed in the Draft Guidelines were subsequently identified as difficult and ambiguous for a triage officer or committee to use to predict mortality risk with any accuracy, and, therefore, any prediction about the patient’s mortality would not be evidence-based.

**TABLE 2:** List of Exclusion Criteria for Ventilator Access for Adult Patients*

Cardiac arrest: unwitnessed arrest, recurrent arrest without hemodynamic stability, arrest unresponsive to standard interventions and measures, trauma-related arrest Irreversible age-specific hypotension unresponsive to fluid resuscitation and vasopressor therapy Traumatic brain injury with no motor response to painful stimulus (i.e., best motor response = 1) Severe burns: where predicted survival ≤ 10% even with unlimited aggressive therapy Any other conditions resulting in immediate or near-immediate mortality even with aggressive therapy

* Adapted from Ontario Health Plan for an Influenza Pandemic Guidelines.

In addition, because the Guidelines modified the definition of survival to be based on the short-term likelihood of survival of the acute medical episode, many of the previously listed medical conditions were no longer applicable. For example, renal dialysis was removed from the exclusion criteria list because it does not serve as a predictor of short-term mortality. Furthermore, to include renal dialysis would necessitate the addition of other resource intensive conditions to the list, many of which are not associated with high mortality. Accordingly, the medical conditions that qualify as exclusion criteria in the Guidelines are limited to those associated with immediate or near-immediate mortality even with aggressive therapy.

Furthermore, the use of a do not resuscitate (DNR) order is explicitly rejected as an exclusion criterion. A DNR order informs health-care professionals that a patient does not wish to receive cardiopulmonary resuscitation (CPR). Because the order does not relate to any other treatment, including ventilator therapy, it is not a reliable proxy for autonomous decision-making during an influenza pandemic.

#### Steps 2 and 3: Sequential Organ Failure Assessment (SOFA) and Periodic Clinical Assessments (Time Trials)

Although the Guidelines reaffirm the use of SOFA to assess a patient’s mortality risk (Table 3) and periodic clinical assessments (time trials) at 48 and 120 h after a patient has begun ventilator therapy to determine if the treatment is effective for the patient (Table 4), they clarify: (1) the SOFA score criteria at the 48- and 120-h assessments and (2) how decisions about whether a patient continues or discontinues ventilator therapy are made.

**TABLE 3:** **Sequential Organ Failure Assessment (SOFA) Score Scale**^**a**^

Variable	0	1	2	3	4
PaO_2_/FiO_2_ mmHg	> 400	< 400	< 300	< 200	< 100
Platelets, x 10^3^/µL	> 150	< 150	< 100	< 50	< 20
(x 10^6^/L)	(> 150)	(< 150)	(< 100)	(< 50)	(< 20)
Bilirubin, mg/dL	< 1.2	1.2 - 1.9	2.0 - 5.9	6.0 - 11.9	> 12
(µmol/L)	(< 20)	(20 - 32)	(33 - 100)	(101 - 203)	(> 203)
Hypotension	None	MABP < 70 mmHg	Dop < 5	Dop 6 - 15 or Epi < 0.1 or Norepi < 0.1	Dop > 15 or Epi > 0.1 or Norepi > 0.1
Glasgow Coma Scale score	15	13 - 14	10 - 12	6 - 9	< 6
Creatinine, mg/dL	< 1.2	1.2 - 1.9	2.0 - 3.4	3.5 - 4.9	> 5
(µmol/L)	(< 106)	(106 - 168)	(169 - 300)	(301 - 433)	(> 434)

Abbreviations: Dop, dopamine; Epi, epinephrine; Norepi, norepinephrine.

a Doses in micrograms per kilogram per minute (administered for at least 1 h). SI units in parentheses. Data adapted from Ferreira et al.^20^

**TABLE 4:** **Mortality Risk Assessment Using SOFA and Time Trials**^a^

Color Code and Level of Access		Assessment of Mortality Risk/Organ Failure^b^		
		Time Trial Periods		
	Initial Assessment	48 h	120 h	Every 48 h
Blue No ventilator provided.^c^ Use alternative forms of medical intervention and/or palliative care or discharge. Reassess if ventilators become available.	Exclusion criterion OR SOFA > 11	Exclusion criterion OR SOFA > 11 OR SOFA 8 – 11 and No change in SOFA score compared with the initial assessment^e^	Exclusion criterion OR SOFA > 11 OR SOFA < 7 and No change in SOFA score compared with the previous assessment	
Red Highest Use ventilators as available.	SOFA < 7 OR Single organ failure^d^	SOFA < 7 and Decrease in SOFA score compared with the initial assessment^f^ OR SOFA < 11 and Decrease in SOFA score compared with the initial assessment^g^	SOFA < 7 and Progressive decrease in SOFA score compared with the previous assessment	
Yellow Intermediate Use ventilators as available.	SOFA 8 – 11	SOFA < 7 and No change in SOFA score compared with the initial assessment	SOFA < 7 and Minimal decrease in SOFA score (< 3 point decrease in previous 72 h) compared with the previous assessment	SOFA < 7 and Minimal decrease in SOFA score (< 3 point decrease in previous 48 h) compared with the previous assessment
Green Use alternative forms of medical intervention or defer or discharge. Reassess as needed.	No significant organ failure AND/OR No requirement for life-saving resources	No longer ventilator dependent / Actively weaning from ventilator		

a Adapted from Ontario Health Plan for an Influenza Pandemic guidelines.

b If a patient develops a condition on the exclusion criteria list at any time during a time trial, change color code to blue. Remove the patient from the ventilator and provide alternative forms of medical intervention and/or palliative care.

c A patient assigned a blue color code is removed from the ventilator and alternative forms of medical intervention and/or palliative care are provided.

d Intubation for control of the airway (without lung disease) is not considered lung failure.

e The patient remains significantly ill.

f These criteria apply to a patient who was placed into the red category at the initial assessment.

g These criteria apply to a patient who was placed into the yellow category at the initial assessment but because a ventilator was available the patient began ventilator therapy.

Previously, the Draft Guidelines did not explain why the SOFA score at 48 h permitted a score of < 11 for the red color code (the highest access for ventilator therapy), when the initial assessment required a SOFA score of < 7 to qualify for the same color code. While most patients who receive ventilator therapy at the initial assessment have a SOFA score < 7, there may be some cases where a patient who was categorized into the yellow color code (SOFA score between 8 and 11 and intermediate access) receives ventilator treatment because ventilators are available and there are no red code patients. Thus, to account for the initial higher (worse) SOFA score of a yellow code patient, the red color criteria permits these patients a higher SOFA ceiling against which their progress is measured at the 48-h assessment (Table 4).

Similarly, at the 120-h assessment, the Guidelines take into account the patient’s SOFA score at the initial assessment. By 120 h, the patient’s health status should have improved such that there should be a significant reduction in SOFA score and any justification for further ventilator treatment by patients in the red color code should be based on a SOFA score of < 7. For patients to be color coded into the yellow category, they should have a SOFA score < 7 (Table 4).

Finally, at both 48- and 120-h assessments, the SOFA score cutoff of < 8 was changed to < 7 for consistency and to reduce confusion regarding overlap of SOFA score cut-offs of the color codes. The Guidelines also clarify that intubation for control of the airway (without lung disease) is not considered lung failure (Table 4).

To continue with ventilator treatment, a patient must demonstrate an improvement in overall health status after receiving ventilator therapy. Patients are not competing against other patients and a triage officer or committee is not permitted to compare patients with one another. Instead, an individual patient’s own health prognosis and trajectory guide the triage decision.

A triage decision is made based on a patient’s SOFA score, which reveals: (1) the overall prognosis estimated by a patient’s clinical indicators, which is indicative of mortality risk by revealing the presence (or likelihood), severity, and number of acute organ failure(s); and (2) the magnitude of improvement or deterioration of overall health (i.e., change in SOFA scores compared with the previous official assessment), which provides additional information about the likelihood of survival with ventilator therapy. The guiding principle for the triage decision is that the more severe a patient’s health condition (i.e., higher the SOFA score) and worsening/no change in mortality risk (i.e., increase or little/no change in the SOFA score), the less likely the patient continues with ventilator therapy. Conversely, the less severe a patient’s health condition (i.e., low SOFA score) and demonstration of improvement with ventilator therapy (i.e., significant decrease in the SOFA score and in mortality risk), the higher the likelihood the patient continues with this form of treatment. Thus, the extent of change in SOFA scores indicates whether a patient is improving, worsening, or experiencing no change in health status and determines whether the patient continues with ventilator therapy.

The Guidelines also more clearly state that the primary difference between the 48- and 120-h assessments is the extent of improvement required to continue to be eligible for ventilator treatment. At 48 h, a patient must exhibit a pattern of significant improvement to be placed in the red color code, the highest access to ventilator therapy. After 120 h, a patient must demonstrate a pattern of *further* significant improvement in health to be placed in the same red color code. The required change in a SOFA score after 120 h of ventilator therapy is expected to be dramatic to qualify for continued use of the ventilator.

Finally, the Guidelines provide guidance on clinical assessments beyond 120 h. A patient who is allocated another time trial for ventilator therapy after the 120-h assessment is reassessed every 48 h using SOFA. Again, the triage decision whether the patient is eligible for continued ventilator treatment is based whether the patient continues to exhibit signs of improvement.

### Secondary Triage Factors: Randomization Process and Young Age

Although all patients will continue to be subject to the clinical scoring criteria enunciated in the Guidelines, when there are no other evidence-based clinical factors to consider to further differentiate patients’ likelihood of survival, a secondary triage factor may be used. If the eligible patient pool consists of only adults, or only red color code patients, a randomization process, such as a lottery, is used each time a ventilator becomes available or a patient needs to be removed from a ventilator. In such cases, a lottery may involve administrative and logistical resources that may be limited during a pandemic. However, because there are no other evidence-based clinical factors available to consider, a lottery might be the fairest option, because it randomly assigns ventilators and all candidates have an equal chance to receive ventilator therapy.

First-come first-served was rejected as a secondary triage factor because it penalizes disadvantaged populations, such as those of lower socio-economic means who might not have access to reliable transportation or minority populations who might initially avoid seeking treatment because of distrust of the health-care system.

The Guidelines again explicitly reject the use of advanced age as a triage criterion, but because of a strong societal preference to protect children, in limited circumstances young age may be considered in a triage decision. Although ventilators should be allocated based on likelihood of survival, in the unique circumstance where all other clinical factors are substantially equal, young age (17 y old and younger) may be an ethically acceptable, secondary (tie-breaking) triage criterion. There may be circumstances where a triage officer or committee makes allocation decisions for both adult and pediatric patients and the available ventilator is a dual-use machine, capable of ventilating either a child or an adult. In such cases, should the clinical evidence indicate that both an adult and pediatric patient have equal (or near equal) likelihoods of survival and there are no other evidence-based clinical factors to further differentiate which patient has a better likelihood of survival, only then may young age be used as a tie-breaker to determine which patient receives or continues with ventilator therapy.

### Other Forms of Medical Intervention and Palliative Care

The Guidelines include a more robust discussion on other forms of medical intervention and palliative care for patients waiting for or who are ineligible for a ventilator. For example, other forms of oxygen delivery, such as nasal cannula, oxygen face masks, BiPAP/CPAP, transtracheal catheters, or other breathing supplements may be used if medically indicated and available. While none of these treatments offer long-term support for a patient with severe influenza, they may sustain a patient long enough for a ventilator to become available. In addition, pharmacological antivirals may provide some benefit to patients.

The Guidelines include a recommendation that hand-held devices, such as bag-valve masks or ambu-bags, should not be permitted at the acute care facility, because such devices may not provide enough oxygen to patients and may contribute to the transmission of the virus. There may be a dearth of health-care staff to manually ventilate these patients, leading to further burden on families if they were responsible for providing this care for the patient.

### Review of Triage Decisions

Triage decisions will inevitably produce dissatisfaction. To address potential legal concerns and ensure that the clinical criteria are applied consistently and fairly, a review process is necessary. In the revised Guidelines, a hybrid system of review—combining limited on-going individual appeals with retrospective, periodic review—that incorporates the advantageous features of both under the constraints of the pandemic, is recommended. Under this system, real-time individual appeals would be limited to procedural or technical injustices (when a withdrawal decision was made without considering all relevant clinical triage criteria) that could remedy a potential injustice before the implementation of a triage decision. Retrospectively, all cases would be reviewed periodically to verify adherence with the Guidelines, thereby enabling evaluation of triage decisions to improve subsequent decisions.

## DISCUSSION

Recently, several entities and states have released or revised recommendations or guidelines regarding ethical and triage considerations when treating critically ill patients during pandemics and disasters,^21-24^ and some are specific to the circumstances of COVID-19.^7,25-31^ The ethical considerations addressed in these recommendations primarily focus on the goal of saving the most lives, mirroring the 2015 Guidelines. A few of these recommendations also emphasize saving those with the most life-years left and/or individuals who have not yet experienced all stages of life,^7,25,26,30,31^ which the 2015 Guidelines explicitly reject as a primary triage criterion (although the 2015 Guidelines incorporate these concepts to justify using young age as a secondary (tie-breaking) triage factor) (see the above section Secondary Triage Factors: Randomization Process and Young Age).

Some of these recommendations deviate from the 2015 Guidelines with regard to the clinical/triage considerations as well. For example, recent recommendations have suggested that exclusion criteria should not be part of a triage protocol, due to their potentially discriminator effects,^25,26,31,32^ and the Office for Civil Rights (OCR) at the United States Department of Health and Human Services (HHS) issued a bulletin^33^ to ensure that institutions do not deny resources based on disability^34^ or advanced age.^7^ While the 2015 Guidelines include exclusion criteria, the list of criteria was revised from the draft 2007 guidelines to only include medical conditions that would fall under the revised definition of (short-term) survival (see the above section Definition of “Survival”). For example, metastatic malignancy with poor prognosis was removed because it is subject to a wide and subjective range of interpretation. Similarly, renal dialysis was also removed as an exclusion criterion because, although this procedure is resource intensive, its use does not affect a person’s short-term survival. The 2007 and the 2015 guidelines explicitly rejected the use of disability or advanced age as exclusion criteria. Despite these efforts, as some states and health-care institutions have moved to not include exclusion criteria in their COVID-19 specific guidelines and recommendations, it may be beneficial for New York to revisit this topic for a future update.

In the context of the COVID-19 pandemic, no state or health-care facility has officially needed to apply the SOFA scoring system to allocate ventilators and, therefore, SOFA continues to be unvalidated for use for triage purposes. To further research into whether SOFA would be a useful tool for triage decisions in the COVID-19 pandemic or other similar scenario, retrospective and prospective studies examining COVID-19 patients in Wuhan, China^35,36^ and New York City,^37^ respectively, have used patients’ clinical data to calculate SOFA scores. While these studies have not yet identified a specific SOFA score range that would place COVID-19 patients into red, yellow, blue, and green color codes, further research into the validity of SOFA scores with COVID-19 will help determine whether SOFA is an appropriate tool in triage protocols.

While the 2015 Guidelines suggest the use of periodic clinical assessments (time trials) at 48 and 120 h after a patient has begun ventilator therapy, followed by subsequent assessments every 48 h, it is clear that these proposed time trials are not applicable to ventilated COVID-19 patients. Numerous studies on ventilated COVID-19 patients have shown that these patients require a median of 10-14 d of mechanical ventilation.^38,39^ Thus, strict adherence to the clinical protocol contained in the 2015 Guidelines, at least in the context of the application of periodic clinical assessments, is not advised.

Like the 2007 Guidelines, the 2015 revisions were issued as guidelines because the static nature of regulations or laws would not be appropriate for clinically detailed recommendations that must be adaptable to changing clinical information about the public health emergency.

The guidelines are a living document, subject to periodic reassessment and adjustment based on societal norms and advances in clinical knowledge. During a pandemic, real-time data collection and analysis should be conducted. The triage process requires regular reassessments of the status of the pandemic, available resources, and of all patients. Information about the pandemic viral strain is vital, and how to treat patients affected by the virus^40,41^ will result in adjusting specific aspects of the triage protocol, such as the length of time trials, SOFA score cutoffs, or adding or removing medical conditions from the exclusion criteria list. Such modifications will ensure that patients receive the best care possible. Furthermore, data collection must include real-time availability of ventilators so that triage decisions are made to allocate resources most effectively.

Furthermore, health-care providers and institutions should be educated about the legal questions that may arise with implementation of the Guidelines, based on the robust legal analysis included in the revised Guidelines.^15,42^ Understanding the potential legal obstacles to effective implementation of the clinical protocol contained in the Guidelines may thereby encourage adherence to them. Under current law, courts may consider the Guidelines to be evidence of the medical standard of care in an influenza pandemic, upon which facilities and providers can rely, thereby providing some liability protection. The Guidelines also recommend that new legislation be enacted to address the medical standard of care specific to the public health emergency, as well as civil and criminal liability protections.

## CONCLUSIONS

This article provides an overview of the changes in the revised New York State Ventilator Allocation Guidelines. The Guidelines rely upon both ethical and clinical evidence-based frameworks in an effort to offer the best possible care under gravely compromised conditions to save the most lives. In developing these Guidelines, the importance of genuine public outreach, education, and engagement cannot be overstated; they are critical to the development of just policies and the establishment of public trust. Finally, although the Guidelines were developed specifically for an influenza pandemic, the framework could be adapted with appropriate modifications for other public health emergencies such as the COVID-19 pandemic that require allocation of scarce resources.

## New York State Task Force on Life and the Law Ventilator Allocation Guidelines Project

*Project Chair*: Susie A. Han, MA, MA

## New York State Task Force on Life and the Law Staff

Susie A. Han, MA, MA; Valerie Gutmann Koch, JD

## New York State Task Force on Life and the Law Members

Howard A. Zucker, MD, JD, LLM (Chair); Karl P. Adler, MD; Adrienne Asch, PhD, MS; Donald P. Berens, Jr., JD; Thomas Berg, PhD, MA; J. David Bleich, PhD; Rock Brynner, PhD, MA; Karen A. Butler, RN, JD; Carolyn Corcoran, JD; Nancy Neveloff Dubler, LLB; Paul J. Edelson, MD; Joseph J. Fins, MD; Francis H. Geer, MDiv; Samuel Gorovitz, PhD; Cassandra E. Henderson, MD; Hassan Khouli, MD; H. Hugh Maynard-Reid, DMin; John D. Murnane, JD; Samuel Packer, MD; Barbara Shack; Robert Swidler, JD; Sally T. True, JD

## Adult Clinical Workgroup Members

Jeffrey T. Berger, MD, Winthrop University Hospital and Stony Brook University School of Medicine; Kenneth Berkowitz, MD, New York University School of Medicine; Irwin Berlin, MD; Cathy Creamer, RN, BSN, MSW, Bellevue Hospital Center; Nancy Neveloff Dubler, LLB, Health and Hospitals Corporation; Joseph J. Fins, MD, Weill Cornell Medical College; Lewis R. Goldfrank, MD, New York University School of Medicine; Jeffrey S. Groeger, MD, Weill Cornell Medical College; John Morley, MD, formerly at New York City Department of Health and Mental Hygiene; Tia Powell, MD, Montefiore-Einstein Center for Bioethics; Neil W. Schluger, MD, Columbia University College of Physicians and Surgeons; Lewis Soloff, MD, formerly at New York City Department of Health and Mental Hygiene; Kristin Stevens, MS, formerly at NYU Langone Medical Center

## References

[1] Wang D, Hu Bo, Hu C, et al. Clinical characteristics of 138 hospitalized patients with 2019 novel coronavirus-infected pneumonia in Wuhan, China. JAMA. 2020;323:1061–1069. 10.1001/jama.2020.1585 PMC7042881 32031570

[2] Yang X, Yu Y, Xu J, et al. Clinical course and outcomes of critically ill patients with SARS-COV-2 pneumonia in Wuhan, China: a single-centered, retrospective, observational study. Lancet Respir Med. 2020;8:475–481. 32105632 10.1016/S2213-2600(20)30079-5 PMC7102538

[3] Guan W, Ni Z, Hu Y, et al. Clinical characteristics of coronavirus disease 2019 in China. N Engl J Med. 2020;382:1708–1720. 32109013 10.1056/NEJMoa2002032 PMC7092819

[4] World Health Organization. Coronavirus disease (COVID-2019) situation reports – 50. March 10, 2020. https://www.who.int/docs/default-source/coronaviruse/situation-reports/20200310-sitrep-50-covid-19.pdf?sfvrsn=55e904fb_2. Accessed March 11, 2020.

[5] Mounk Y. The extraordinary decisions facing Italian doctors. The Atlantic. March 11, 2020 https://www.theatlantic.com/ideas/archive/2020/03/who-gets-hospital-bed/607807/. Accessed March 11, 2020.

[6] Remuzzi A, Remuzzi G. COVID-19 and Italy: what next? Lancet. 2020;39S:1225–1228. 10.1016/S0140-6736(20)30627-9 PMC7102589 32178769

[7] Italian Society of Anesthesia. Analgesia, Resuscitation and Intensive Care (Società Italiana di Anestesia Analgesia Rianimazione e Terapia Intensiva [SIAARTI]). Clinical Ethics Recommendations for the Allocation of Intensive Care Treatment, in Exceptional, Resource-Limited Circumstances, Version n.1, March 16, 2020 http://www.siaarti.it/SiteAssets/News/COVID19%20-%20documenti%20SIAARTI/SIAARTI%20-%20Covid-19%20-%20Clinical%20Ethics%20Reccomendations.pdf. Accessed March 26, 2020.

[8] Kinross P, Suetens C, Gomes Dias J, et al. Rapidly increasing cumulative incidence of coronavirus disease (COVID-19) in the European Union/European Economic Area and the United Kingdom, 1 January to 15 March 2020. Euro Surveill. 2020;25(11):2000285. 10.2807/1560-7917.ES.2020.25.11.2000285 PMC7096777 32186277

[9] McKinley J. New York City Region is now an epicenter of the coronavirus pandemic. N.Y. Times. March 22, 2020 https://www.nytimes.com/2020/03/22/nyregion/Coronavirus-new-York-epicenter.html. Accessed March 26, 2020.

[10] Rosenthal BM, Goldstein J. N.Y. May Need 18,000 Ventilators Very Soon. It is Far Short of That. N.Y. Times. March 17, 2020 https://www.nytimes.com/2020/03/17/nyregion/ny-coronavirus-ventilators.html?action=click&module=RelatedLinks&pgtype=Article. Accessed March 26, 2020.

[11] Rubinson L, Hick JL, Hanfling DG, et al. Definitive care for the critically ill during a disaster: a framework for optimizing critical care surge capacity. Chest. 2008;133(Suppl):18S–31S. 18460504 10.1378/chest.07-2690 PMC7094361

[12] Truog RD, Mitchell C, Daley GQ. The toughest triage – allocating ventilators in a pandemic. N Engl J Med. 2020;382:1973–1975. 32202721 10.1056/NEJMp2005689

[13] Powell T, Christ KC, Birkhead GS. Allocation of ventilators in a public health disaster. Disaster Med Pub Health Prep. 2008;2:20–26. 18388654 10.1097/DMP.0b013e3181620794

[14] New York State Workgroup on Ventilator Allocation in an Influenza Pandemic. Allocation of ventilators in an influenza pandemic: planning document. March 15, 2007 https://www.cidrap.umn.edu/sites/default/files/public/php/196/196_guidance.pdf. Accessed July 11, 2020.

[15] New York State Task Force on Life and the Law and New York State Department of Health. Ventilator allocation guidelines. November 2015 https://www.health.ny.gov/regulations/task_force/reports_publications/docs/ventilator_guidelines.pdf. Accessed March 10, 2020.

[16] World Health Organization. WHO Director-General’s Opening Remarks at the Media Briefing on COVID-19. March 11, 2020 https://www.who.int/dg/speeches/detail/who-director-general-s-opening-remarks-at-the-media-briefing-on-covid-19---11-march-2020. Accessed March 11, 2020.

[17] Kaji A, Koenig KL, Bey T. Surge capacity for healthcare systems: a conceptual framework. Acad Emerg Med. 2006;13:1157–1159. 16968688 10.1197/j.aem.2006.06.032

[18] Christian MD, Hawryluck L, Wax RS, et al. Development of a triage protocol for critical care during an influenza pandemic. CMAJ. 2006;175:1377–1381. 17116904 10.1503/cmaj.060911 PMC1635763

[19] Ontario Health Plan for an Influenza Pandemic (OHPIP) Working Group on Adult Critical Care Admission, Discharge, and Triage Criteria. *Critical Care During a Pandemic* April 2006 http://www.cidrap.umn.edu/sites/default/files/public/php/21/21_report.pdf. Accessed March 10, 2020.

[20] Ferreira FL, Bota DP, Bross A, et al. Serial evaluation of the SOFA score to predict outcome in critically ill patients. JAMA. 2001;286:1754–1758. 11594901 10.1001/jama.286.14.1754

[21] Biddison LD, Berkowitz KA, Courtney B, et al. Ethical considerations: care of the critically ill and injured during pandemics and disasters: CHEST consensus statement. Chest. 2014;146(Suppl):e145S–e155S. 25144262 10.1378/chest.14-0742

[22] Christian MD, Spring CL, King MA, et al. Triage: care of the critically ill and injured during pandemics and disasters: CHEST consensus statement. Chest. 2014;146(Suppl):e61S–e74S. 25144591 10.1378/chest.14-0736 PMC7127536

[23] Baker M, Fink S. At the top of the Covid-19 curve, how do hospitals decide who gets treatment? N.Y. Times. March 31, 2020 https://www.nytimes.com/2020/03/31/us/coronavirus-covid-triage-rationing-ventilators.html?searchResultPosition=6. Accessed March 31, 2020.

[24] Daugherty Biddison, EL, Faden R, Gwon HS, et al. Too many patients… a framework to guide statewide allocation of scarce mechanical ventilation during disasters. Chest. 2019;155:848–854. 30316913 10.1016/j.chest.2018.09.025

[25] Pennsylvania Department of Health and The Hospital + Healthsystem Association of Pennsylvania. Interim Pennsylvania Crisis Standards of Care for Pandemic Guidelines. March 22, 2020 https://int.nyt.com/data/documenthelper/6850-pennsylvania-triage-guidelines/02cb4c58460e57ea9f05/optimized/full.pdf. Accessed May 22, 2020.

[26] University of Pittsburgh, Department of Critical Care Medicine, School of Medicine. Allocation of scarce critical care resources during a public health emergency. April 15, 2020 https://ccm.pitt.edu/sites/default/files/UnivPittsburgh_ModelHospitalResourcePolicy_2020_04_15.pdf. Accessed May 18, 2020.

[27] Montana Department of Public Health and Human Services and the Montana Hospital Association. Scarce resource management & crisis care guidance. https://mtha.org/wp-content/uploads/2020/04/Montana-Crisis-Care-Guidance-Final.pdf. Accessed May 22, 2020.

[28] Maves RC, Downar J, Dichter JR, et al. Triage of scarce critical care resources in COVID-19 an implementation guide for regional allocation: an expert panel report of the task force for mass critical care and the American College of Chest Physicians. *Chest* Published online April 11, 2020 10.1016/j.chest.2020.03.063. Accessed May 18, 2020. PMC7151463 32289312

[29] Swiss Academy of Medical Sciences (SAMWASSM). COVID-19 pandemic: triage for intensive-care treatment under resource scarcity. Swiss Med Wkly. 2020;150:w20229. https://smw.ch/article/doi/smw.2020.20229. Accessed May 18, 2020. 10.4414/smw.2020.20229 32208495

[30] Emanuel EJ, Persad G, Upshur R, et al. Fair allocation of scarce medical resources in the time of COVID-19. N Engl J Med. 2020;382:2049–2055. 32202722 10.1056/NEJMsb2005114

[31] White DB, Lo B. A framework for rationing ventilators and critical care beds during the COVID-19 pandemic. JAMA. 2020;323:1773–1774. 10.1001/jama.2020.5046 32219367

[32] Auriemma CL, Molinero AM, Houtrow AJ, et al. Eliminating categorical exclusion criteria in crisis standards of care frameworks. *Am J Bioeth* Published online May 18, 2020. doi/full/10.1080/15265161.2020.1764141. Accessed May 22, 2020. 10.1080/15265161.2020.1764141 PMC7387214 32420822

[33] US Department of Health and Human Services, Office for Civil Rights. Bulletin: Civil rights, HIPAA, and the coronavirus disease 2019 (COVID-19). March 28, 2020, Updated April 3, 2020. https://www.hhs.gov/sites/default/files/ocr-bulletin-3-28-20.pdf?fbclid=IwAR351WokrC2uQLIPxDR0eiAizAQ8Q-XwhBt_0asYiXi91XW4rnAKW8kxcog. Accessed May 22, 2020.

[34] US Department of Health and Human Services, Office for Civil Rights. OCR reaches early case resolution with Alabama after it removes discriminatory ventilator triaging guidelines. April 8, 2020 https://www.hhs.gov/about/news/2020/04/08/ocr-reaches-early-case-resolution-alabama-after-it-removes-discriminatory-ventilator-triaging.html. Accessed May 22, 2020.

[35] Zhou F, Yu T, Du R, et al. Clinical course and risk factors for mortality of adult inpatients with COVID-19 in Wuhan, China: a retrospective cohort study. Lancet. 2020;39S:1054–1062. 10.1016/S0140-6736(20)30566-3 PMC7270627 32171076

[36] Tang X, Du R, Wang R, et al. Comparison of hospitalized patients with ARDS caused by COVID-19 and H1N1. *Chest* Published online March 26, 2020 https://journal.chestnet.org/article/S0012-3692(20)30558-4/pdf. Accessed May 22, 2020. 10.1016/j.chest.2020.03.032 PMC7151343 32224074

[37] Cummings MJ, Baldwin MR, Abrams D, et al. Epidemiology, clinical course, and outcomes of critically ill adults with COVID-19 in New York City: a prospective cohort study. *Lancet* Published online May 19, 2020 https://www.thelancet.com/pdfs/journals/lancet/PIIS0140-6736(20)31189-2.pdf. Accessed May 22, 2020. 10.1016/S0140-6736(20)31189-2 PMC7237188 32442528

[38] Bhatraju PK, Ghassemieh BJ, Nichols M, et al. Covid-19 in critically ill patients in the Seattle region- case series. N Engl J Med. 2020;382:2012–2022. 32227758 10.1056/NEJMoa2004500 PMC7143164

[39] Hur K, Price CPE, Gray EL, et al. Factors associated with intubation and prolonged intubation in hospitalized patients with COVID-19. *Otolaryngol Head Neck Surg.* Published online May 19, 2020 10.1177/0194599820929640. Accessed May 18, 2020. PMC7240317 32423368

[40] World Health Organization. Clinical Management of Severe Acute Respiratory Infection (SARI) when COVID-19 disease is suspected, interim guidance. May 27, 2020 https://apps.who.int/iris/bitstream/handle/10665/332196/WHO-2019-nCoV-clinical-2020.5-eng.pdf?sequence=1&isAllowed=y. Accessed June 1, 2020.

[41] Niederman MS, Richeldi L, Chotirmall SH, et al. Rising to the challenge of the novel SARS-coronavirus-2 (SARS-CoV-2): advice for pulmonary critical care and an agenda for research. Am J Respir Crit Care Med. 2020;201:1019–1022. 32203671 10.1164/rccm.202003-0741ED PMC7193856

[42] Koch VG, Roxland BE. Unique proposals for limiting legal liability and encouraging adherence to ventilator allocation guidelines in an influenza pandemic. DePaul J Health Care Law. 2013;14(3):467–501.

